# Liquid-Phase Packaging of a Glucose Oxidase Solution with Parylene Direct Encapsulation and an Ultraviolet Curing Adhesive Cover for Glucose Sensors

**DOI:** 10.3390/s100605888

**Published:** 2010-06-09

**Authors:** Seiichi Takamatsu, Hisanori Takano, Nguyen Binh-Khiem, Tomoyuki Takahata, Eiji Iwase, Kiyoshi Matsumoto, Isao Shimoyama

**Affiliations:** The University of Tokyo, 7-3-1 Hongo, Bunkyo-ku, Tokyo, Japan; E-Mails: takano@leopard.t.u-tokyo.ac.jp (H.T.); khiem@leopard.t.u-tokyo.ac.jp (N.B.-K.); takahata@leopard.t.u-tokyo.ac.jp (T.T.); iwase@leopard.t.u-tokyo.ac.jp (E.I.); matsu@leopard.t.u-tokyo.ac.jp (K.M.); isao@leopard.t.u-tokyo.ac.jp (I.S.)

**Keywords:** glucose sensor, glucose oxidase, Parylene, packaging

## Abstract

We have developed a package for disposable glucose sensor chips using Parylene encapsulation of a glucose oxidase solution in the liquid phase and a cover structure made of an ultraviolet (UV) curable adhesive. Parylene was directly deposited onto a small volume (1 μL) of glucose oxidase solution through chemical vapor deposition. The cover and reaction chamber were constructed on Parylene film using a UV-curable adhesive and photolithography. The package was processed at room temperature to avoid denaturation of the glucose oxidase. The glucose oxidase solution was encapsulated and unsealed. Glucose sensing was demonstrated using standard amperometric detection at glucose concentrations between 0.1 and 100 mM, which covers the glucose concentration range of diabetic patients. Our proposed Parylene encapsulation and UV-adhesive cover form a liquid phase glucose-oxidase package that has the advantages of room temperature processing and direct liquid encapsulation of a small volume solution without use of conventional solidifying chemicals.

## 1. Introduction

The number of diabetic patients has recently been increasing, reaching up to 180 million people world-wide according to a WHO report, so there has been high demand for blood glucose measurement methods for use in the diagnosis of diabetes [1]. Glucose sensors consist of enzymes that catalyze a selective chemical reaction with glucose and require the use of sensing transducers comprised of electrochemical electrodes. Many important studies investigating various components of such sensors have been conducted [3–6]. Among the components of interest, the process of packaging an enzymatic solution (*i.e.*, glucose oxidase) onto electrochemical electrodes is important, especially considering the need for disposable sensors [7–14]. To properly package glucose oxidase solution, two approaches have been investigated: directly packaging the solution and solidifying the solution [8,10]. Direct packaging of the glucose oxidase solution with plastic or other materials has the advantage of avoiding the addition of specific chemicals to solidify the liquid (*i.e.*, cross-linker, gel), which could affect the properties of the glucose oxidase enzyme [2–4]. However, liquid phase packaging has not been achieved to date because this approach requires that a small volume of the solution (1 μL) be packaged without denaturing the biomaterial-based enzyme.

Glucose oxidase is derived from *Aspergillus niger* and preserved as a solution. The reactivity of the oxidase to glucose is affected by high temperatures (>50 °C) and alcohols or other chemicals [2–4]. Existing micro-packaging techniques used to package small volumes of glucose oxidase solution include plastic sealing, anodic bonding, and ultrasonic bonding [15,16]. Importantly, most of these methods use high temperatures (>150 °C) to melt and modify the surface of plastic, glass and silicon substrates for bonding. In a previous study [13], a micro-package was designed to inject the solution after the bonding process was completed to avoid heating of the enzyme solution upon wafer bonding. A package made from a silicon wafer that contained chambers and holes was bonded to the sensor wafer. The necessary volume of glucose oxidase solution could be measured by pouring it through holes; however, the holes needed to be sealed afterwards to store the solution. To achieve a lower bonding temperature, another group [17] used Parylene-Parylene bonding and directly sealed a liquid solution in a Parylene-coated silicon-package and sensor wafer. Although the bonding temperature was lower than that of the other bonding process, 180 °C was required for Parylene-Parylene adhesion, and the solvent for the glucose oxidase solution (*i.e.*, water) evaporated upon sealing. Therefore, the packaging of liquid glucose oxidase still relies on high temperature heating and evaporation of solvent, which are undesirable processing steps.

**Figure 1.**
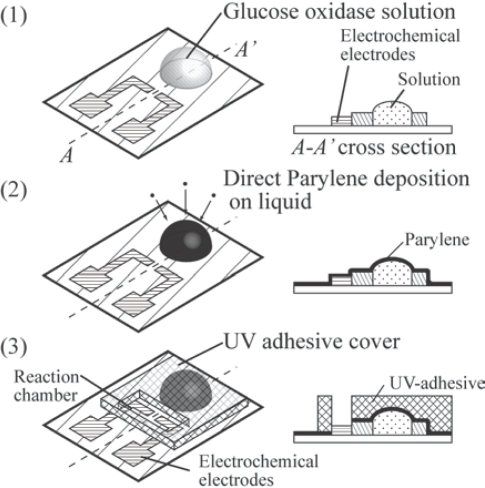
Concept and structure.

In the present study, we have developed a packaging process that involves Parylene encapsulation of glucose oxidase solution and use of a UV-adhesive cover to enable low temperature packaging (Figure 1). At room temperature, Parylene can be deposited on the glucose oxidase solution because Parylene vapor is polymerized on the solution without heating, thereby forming a capsule [Figure 1(2)]. A UV-adhesive structure can also be constructed at room temperature because the adhesive is cured under UV illumination [Figure 1(3)]. To confirm the effectiveness of the proposed package, the package was characterized in terms of its encapsulation of glucose oxidase solution and unsealing of the capsule. Glucose sensing by the packaged glucose oxidase solution was assessed to demonstrate the applicability of the package for glucose sensors.

## 2. Experimental

### 2.1. Structure of the Package Containing the Glucose Oxidase Solution

The package was placed on the two electrochemical electrodes [Figure 1(3)]. The package consisted of a Parylene capsule of glucose oxidase solution and a UV-adhesive cover that constituted the cover to cover the Parylene capsule and the reaction chambers to react glucose oxidase with glucose solution. The dimensions of the sensor are approximately 10 mm × 10 mm. The size is similar to that of standard disposable glucose sensor chips [4], in which the sensor consists of two electrochemical electrodes and an enzymatic gel or polymer. 1 μl-solution measured with a micro-pipette was dropped on 2.5-mm diameter circle and encapsulated by Parylene. The UV-adhesive cover overlays the capsule. The height of the cover is 2 mm, which is sufficient to cover the capsule. The reaction chamber for glucose and the glucose oxidase solution occupies an area of 1 mm × 3 mm. The gold electrodes (area of 1 mm × 1 mm) are located under the reaction chamber.

The standard amperometric method was used because its sensing time is fast and conventional. The glucose-glucose oxidase chemical reaction is as follows:

$$\text{D-glucose}+H_{2}O+O_{2}D\;\overset{\mathrm{Clucose}\;\mathrm{oxidase}}{\to }\;\;\text{gluconic acid}+\;\;H_{2}O_{2}$$

The generated hydrogen peroxide was detected by applying an electric potential to the two electrochemical electrodes. The reaction on the electrodes includes the following redox electrochemical reactions:

$$\begin{array}{c} \text{Anode}:H_{2}O_{2}\to O_{2}+{2H}^{+}+{2e}^{-}\\ \text{Cathode}:\;1/2\;O_{2}+{2H}^{+}+{2e}^{-}\to H_{2}O \end{array}$$

The reaction formula describes current flow from the cathode to the anode according to the decomposition of hydrogen peroxide. The gold electrochemical electrodes were used to detect the resultant current. The current indicated the glucose concentration.

### 2.2. Preparation of Glucose Oxidase Solution and Glucose Solution

Powdered glucose oxidase (G0050, Tokyo Kasei Kogyo) was diluted in solvent to a concentration of 500 units of glucose oxidase per 38.2 mL of solvent. In the reaction of glucose and glucose-oxidase, the pH of the solvent affects the reaction rate and the resultant electric current during the electrochemical measurement. The optimal temperature is 30–40 °C, and the optimal pH is 4–7. Phosphate-buffered saline (pH = 6.7; product number 70011–044, GIBCO) was used in the solvent to maintain a constant pH. After the glucose oxidase powder was dissolved in phosphate-buffered saline, an equivalent volume of 1-ethyl-3-methylimidazolium ethyl sulfate ionic solution [19,20] was added, and the mixture was stored overnight to allow for evaporating water in buffer solution. The glucose solution used in the glucose concentration experiment was prepared by mixing d-glucose (Kanto Kagaku) and water to final concentrations of 0.1, 1, 10, and 100 mM, which covers the range of glucose observed in diabetic patients.

### 2.3. Fabrication of Electrochemical Electrodes

**Figure 2.**
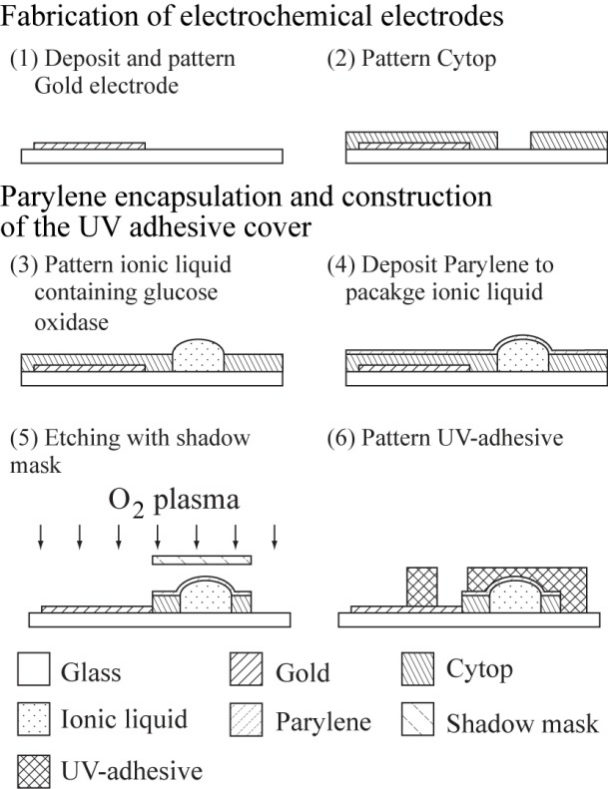
Fabrication process.

Figure 2 shows the fabrication process of the proposed package. Electrochemical electrodes are fabricated on a glass slide with a thickness of 1.3 mm. A 5-nm thick chromium adhesion layer and 100-nm thick gold electrode were deposited on the slide by a thermal evaporator (SANYU Corporation SCV-700).

The electrodes were patterned using an AZP 4620 photoresist as a mask and etched with standard etchants used in microfabrication [Figure 2(1)]. On the electrode, a hydrophobic material, namely a fluoropolymer (Cytop 809, Asahi glass), was coated and patterned to define the area that must not be covered in glucose oxidase solution. Cytop was spin-coated on the glass slide at a speed of 500 rpm and annealed at a temperature of 185 °C for 30 min. For patterning, an AZP 4620 photoresist was used as a mask and etched with an oxygen-plasma etcher (SAMCO compact etcher) at a flow rate of 5 mL/min for 2 min. Because the photoresist reduced the hydrophobicity of Cytop, the film was re-annealed at 185 °C for 10 min after removal of the photoresist using acetone and ethanol [Figure 2(2)].

### 2.4. Parylene Encapsulation and Construction of the UV Adhesive Cover

One μL of the prepared glucose oxidase solution that was measured by micropipette was coated on the desired area of substrate, which had been defined on the substrate due to the wettability of the hydrophilic glucose oxidase solvent which was the ionic liquid 1-ethyl-3-methylimidazolium ethyl sulfate [Figure 2 (3)] described in the section of preparation of glucose oxidase solution and glucose solution. A Parylene film was formed on the patterned ionic liquid by room-temperature chemical vapor deposition (CVD) using a coat of PDS2010 (Japan Parylene) as shown in Figure 2(4). Although CVD requires a vacuum at a vapor pressure of 2.7 Pa [18], the ionic liquid 1-ethyl-3-methyl-imidazolium ethyl sulfate used did not evaporate because of its vapor pressure is almost zero [19,20]. The Parylene on the electrochemical electrodes was etched by oxygen plasma through a shadow mask [Figure 2(5)].

On the Parylene capsule, a cover with a 2-mm height was constructed using UV adhesive. A highly viscous adhesive (NOA68) is easy to cast with a large height (2 mm), so it can be used to make covers that are several millimeters long. As for the patterning process, a structure made of a UV-curable adhesive can be exposed through a photomask by UV illumination, developed in acetone and rinsed in ethanol [Figure 2(6)]. Therefore, heating is not required to create a complex UV-adhesive structure, whereas standard thick photoresists such as SU-8 used to form micro to millimeter-scale structures require destructive heating (>100 °C) for drying of the resist solvent and curing the photoresist polymer.

### 2.5. Measurement of the Glucose Concentration

**Figure 3.**
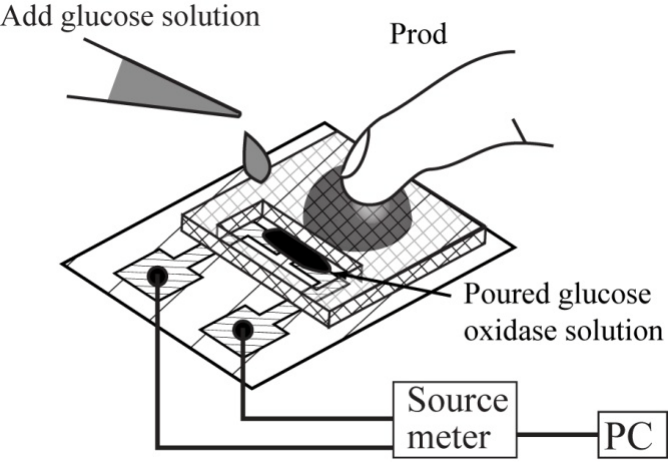
Experimental setup of glucose sensing with a packaged glucose oxidase solution.

To evaluate the utility of the package for glucose concentration sensing, a glucose sensing experiment was conducted. Figure 3 shows the experimental setup. Electrochemical electrodes were connected to source meter which was controlled by a PC and Labview software. In the experiment, packaged glucose oxidase solution was poured to the reaction chamber by prodding the UV-adhesive cover. Then, the glucose solution for testing was added to reaction chamber and reacted with glucose oxidase solution. The resultant current described in the section of the structure of the package containing the glucose oxidase solution was measured with source meter by applying electric potential of 0.7 V between electrochemical electrodes.

## 3. Results and Discussion

### 3.1. Encapsulation and Unsealing of the Package

**Figure 4.**
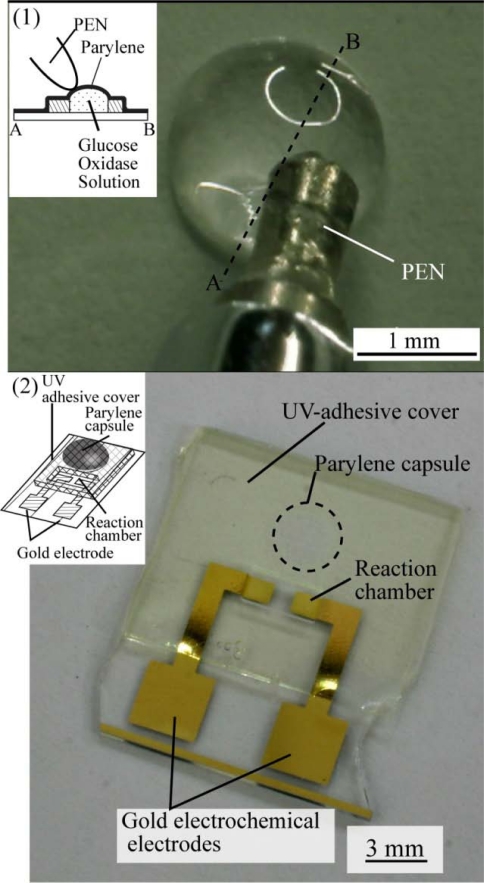

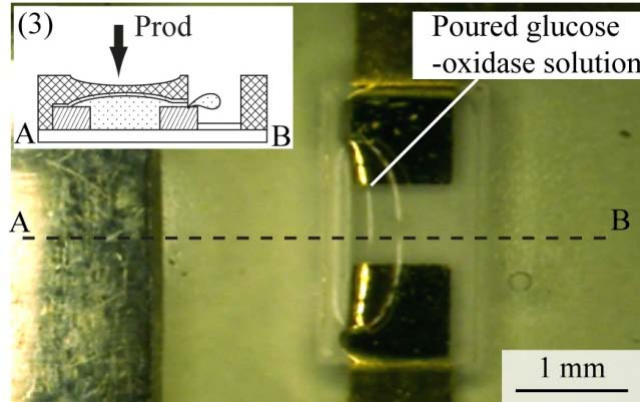
Fabricated device.

Figure 4(1) shows the glucose oxidase solution on electrochemical electrodes that were encapsulated with Parylene.

The top part of the hemispherical shape is the glucose oxidase liquid capsule. Prodding of the Parylene film with a pen did not result in penetration of the film, which indicates that the glucose oxidase solution was fully encapsulated by the Parylene film. Figure 4(2) shows how a structure made of a UV adhesive could be constructed on the Parylene capsule. The UV adhesive covered both the Parylene capsule and the reaction chamber on electrochemical electrodes. The capsule could be unsealed by prodding, as shown in Figure 4(3). The encapsulated solution was then poured into the reaction chamber.

### 3.2. Demonstration of Glucose Sensing with a Packaged Glucose Oxidase Solution

The required range of glucose sensing for disposable glucose sensor chips is 1 to 100 mM, which covers the range of glucose concentrations found in human blood [4]. In this section, we demonstrate glucose sensing in this range using the packaged glucose oxidase solution.

**Figure 5.**
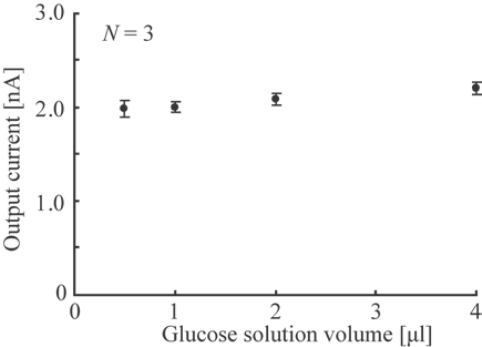
Relationship between the glucose solution volume and the output current.

The relationship between the volumes of added glucose solution and output currents was examined. In principle, the output current is defined by the concentration of glucose in the solution, not by its volume. The added volumes varied from 0.5 to 2 μL for a constant glucose concentration (100 mM), and the resultant currents were measured. Figure 5 shows that the current generated by the reaction did not depend on the volume of the glucose solution. The difference in the current is less than 5% over the solution range of 0.5 to 2 μL. This result indicates that the glucose solution can be added to sensors without considering the glucose solution volume.

**Figure 6.**
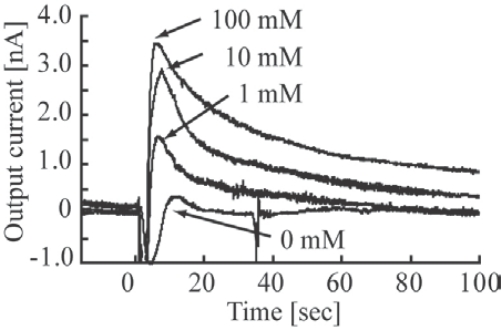
Relationship between the time and current at different concentrations of glucose.

The relationship between glucose concentrations and the resultant currents was examined. An experiment that assessed glucose concentration sensitivity was performed following addition of 1 μL of glucose solution at concentrations of 0.1, 1, 10, and 100 mM to the reaction chamber of the package. The time response waveforms of the generated currents are shown in Figure 6. The output currents increase according to the concentration of glucose in the solutions; however, the reaction time stays nearly constant.

**Figure 7.**
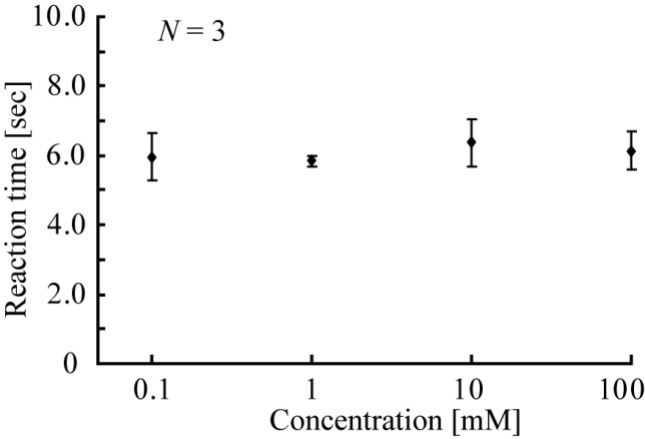
Relationship between glucose concentration and reaction time.

Figure 7 shows the relationship between target glucose concentrations and reaction times. Although the x-axis indicates the logarithm of the concentration, the reaction times are nearly the same (approximately six seconds). The errors in the reaction times for different concentrations of glucose are approximately 5%, and the concentration did not affect the reaction time. The reaction time with the packaged liquid glucose oxidase solution was found to be 6 s.

**Figure 8.**
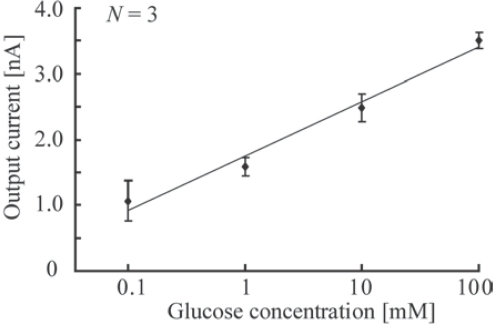
Relationship between glucose concentration and current.

The relationship between the glucose concentration and current is shown in Figure 8. The output current was measured three times for each concentration, and the average current was plotted with error bars. The y-axis is the logarithm of the generated current, while the x-axis is the concentration. The logarithm of the current is proportional to the concentration. The determined coefficient factor between the concentration and the current is approximately 0.98. This figure suggests that the proposed glucose sensor can detect glucose concentrations ranging from 0.1 to 100 mM, which is the suitable range for the diagnosis of diabetic patients.

## 4. Conclusions

A low-temperature packaging process to seal glucose oxidase solution in the liquid phase without denaturation was developed using a package of Parylene-encapsulated glucose oxidase solution and a UV-adhesive cover. The proposed Parylene encapsulation can package glucose oxidase solution at room temperature, and the UV adhesive (NOA68) can be constructed by patterning and curing by UV illumination instead of heating.

During fabrication of the developed package, a glucose oxidase solution including a 1-ethyl-3-methylimidazolium ethyl sulfate ionic liquid was patterned on a hydrophobic-hydrophilic electrode modified with a fluoropolymer (Cytop) according to wettability. A 1.5-μm thick Parylene layer was directly deposited onto a small volume (1 μL) of glucose oxidase solution through chemical vapor deposition. On the Parylene film, a cover and reaction chamber with an area of 1 mm × 3 mm and height of 2 mm was constructed from UV-curable adhesive (NOA68) through photolithography. The proposed package was processed at room temperatures to avoid denaturation of the glucose oxidase enzyme. The glucose oxidase solution was packaged in the fabricated structure and could be easily opened by prodding.

We have demonstrated glucose sensing with a packaged glucose-oxidase solution. The output current was found to be independent of the volume of glucose solution over a range of 0.5 to 2 μL. The time required to react the glucose with the glucose oxidase solution was 6 s. Sensitivity to the glucose concentration was achieved over a range of 0.1 to 100 mM glucose, which is the range of glucose concentrations seen in diabetic patients.

Our proposed Parylene encapsulation process and UV-adhesive cover allows liquid phase glucose-oxidase packaging at room temperature and direct liquid encapsulation of small volumes of solution without use of conventional solidifying chemicals. Future work concerning this package will include quantitatively evaluating the decrease of glucose oxidase function during the fabrication process, testing for long-term stability for storage and modification of the package to create other types of sensing electrodes, such as ion sensitive-field effect transistors [10] and organic electrochemical transistors [5].
